# Cognitive Manifestations of Racemose Neurocysticercosis: A Two Case Report

**DOI:** 10.1002/alz70857_105260

**Published:** 2025-12-25

**Authors:** Marcelo Jobet, Veronica Perez, Oscar Arias, Sebastian Bravo, Rodrigo A Santibanez

**Affiliations:** ^1^ Memory Clinic, Neurology Service, Complejo Asistencial Doctor Sótero del Río, Santiago, Chile; ^2^ Neurology Department, Faculty of Medicine, Pontificia Universidad Católica de Chile, Santiago, Chile; ^3^ Radiology Department, Faculty of Medicine, Pontificia Universidad Católica de Chile, Santiago, Chile

## Abstract

**Background:**

Neurocysticercosis (NCC) is a central nervous system infection caused by the larval form of the tapeworm Taenia solium, endemic in Latin America and Asia. It can present as intraparenchymal or racemose (rNCC) forms, with symptoms including epilepsy, headaches, and intracranial hypertension. We present two cases of rNC from Chile with significant cognitive symptoms.

**Method:**

First case is a 79‐year‐old male farmer with suspected normal pressure hydrocephalus (NPH). Symptoms included progressive forgetfulness, disorientation, gait disorder, and headache over four years. Investigations included a magnetic resonance of the brain (MRI) that revealed multiple subarachnoid cysts and hydrocephalus compatible with rNCC. Cerebrospinal fluid (CSF) analysis showed pleocytosis, hyperproteinorrhacia, and hypoglycorrhachia. Blood Enzyme‐Linked ImmunoSorbent Assay (ELISA) and CSF Western blot (WB) confirmed cysticercosis. He was treated with corticosteroids, albendazole, and praziquantel over a month. Follow‐up neuroimaging showed decreased ventricular widening and cysts volume. Second case is a 58‐year‐old male from Santiago who presented with confusion, agitation, disconnection episodes, and headache over two months. An MRI revealed signs of basal meningitis and interhemispheric and anterior cistern cysts with leptomeningeal enhancement. CSF had pleocytosis, hyperproteinorrhacia, and hypoglycorrhachia. ELISA and WB confirmed cysticercosis. He began treatment with albendazole and corticosteroids, completing 30 days. Follow‐up MRI confirmed almost complete resolution of the lesions.

**Result:**

Both cases presented with prominent cognitive features, with the first case resembling NPH and the second case presenting as a subacute confusional state/rapidly progressive dementia. Corticosteroids plus antiparasitic drugs led to significant improvements in MRI abnormalities, with no major complications. Although the cognitive symptoms showed considerable improvement, they did not completely resolve.

**Conclusion:**

Although rare, rNCC should be considered as a differential diagnosis for a wide range of neurological conditions in regions where NCC is common. Atypical clinical findings and headache should be regarded as warning signs. T2 and T1 gadolinium‐enhanced MRI sequences were essential in assessing these cases. CSF showed a characteristic profile, and blood ELISA and CSF WB were able to confirm the diagnosis. Treatment with antiparasitic drugs combined with corticosteroids appears to be effective and well tolerated. Further research is necessary to improve the diagnosis and treatment of rNCC.

